# Influence of Austempering of As-Cast Medium Carbon High-Silicon Steel on Wear Resistance

**DOI:** 10.3390/ma14247518

**Published:** 2021-12-08

**Authors:** Marko Sedlaček, Grega Klančnik, Aleš Nagode, Jaka Burja

**Affiliations:** 1 Institute of Metals and Technology, Lepi Pot 11, 1000 Ljubljana, Slovenia; jaka.burja@imt.si; 2 Prolabor d.o.o., Podvin 20, 3310 Žalec, Slovenia; grega.klancnik@prolabor.si; 3 Department for Materials and Metallurgy, Faculty of Natural Sciences and Engineering, University of Ljubljana, Aškerčeva 12, 1000 Ljubljana, Slovenia; ales.nagode@omm.ntf.uni-lj.si

**Keywords:** austempering, lower bainite, wear, impact toughness, hardness

## Abstract

The aim of this study was to evaluate the effect of austempering compared to quenching and low-temperature tempering on wear resistance of an as-cast medium carbon high-silicon steel intended for rock breaking. Austempering was done by isothermal holding at 270, 300 and 350 °C in molten salt baths, while quenching was done in water. The austempering treatments resulted in microstructural combinations of bainite and martensite. The isothermal holding at 270 °C resulted in bainite and self-tempered martensite, while isothermal holdings at 300 and 350 °C resulted in bainite and untempered martensite. The two quench and temper treatments resulted in tempered martensite. In general austempering resulted in lower hardness values when compared to quenching and tempering but higher impact toughness. The wear resistance was best for quenching and low temperature tempering, followed by austempering at 270 °C, but at slightly lower hardness and 25% higher impact toughness. The other two austempering treatments resulted in worse wear resistance.

## 1. Introduction

Wear of surfaces is present in a variety of engineering applications and has both economic and technical consequences. From an economic perspective, the cost caused by friction and wear is estimated in the range of 1% to 4% of the gross national product of industrialized countries [1]. The effect of abrasion wear is particularly evident in industrial fields such as agriculture, mining, mineral processing and earthmoving work. This is especially true for hydraulic breakers used by excavators. Excavators play and important role in mining and open pit quarries so their productivity has been studied [2,3]. In our case, the material used for the tooth for rock breaking is studied. These larger parts with complex design are produced by gravitational casting. This means that the hot working processes (forging and/or rolling) that are ordinarily employed for final metallurgical and related mechanical characteristics are not used in this study. While close-to-shape casting enables time efficient production of complex shape products, it also means that the heat treatment and carefully chosen chemical composition is the only option for microstructural design. However, it is necessary to obtain a fully sound casting for proper heat treatment response. Excessive presence of shrinkage, gas porosity and slag entrapment etc. can substantially degrade mechanical properties and can cause premature on-field failure meaning that internal quality is highly important also for heat treatment sensibility study. Wear is also one of the key problems in many types of engineering components as being a very important factor in determining the life expectancy of certain components.

Material strength (hardness) and toughness are the main properties that have been observed to increase wear resistance [4]. In general, the abrasive wear rate is proportional to the hardness of the material, which is especially true for metals. Development of high wear resistant steels, therefore, mostly focuses on ensuring high hardness on the basis of a martensitic matrix. However, in certain cases the material toughness has been found to have a major influence in terms of wear resistance [4,5]. The brittleness of martensite may, in some cases, cause a lower wear resistance, even though the hardness of the material is very high. Different experimental findings show that just a high hardness matrix does not guarantee high wear resistance [6].

Properties of cast steels can be modified with the chemical composition [7] and/or heat treatment [8]. The conventional quenching approach is composed from heating to the temperature of austenitization, quenching and tempering. During austenitization, the matrix is transformed into austenite. After a certain retention time at the austenitization temperature, to ensure a fully austenitic microstructure, the steel is then quenched. The rapid cooling in a quenching medium (typically in oil or water) ensures the nonequilibrium transformation of austenite to martensite. The resulting martensite is hard and brittle, and must therefore be tempered to achieve the desired properties, thus lowering hardness and increasing toughness. However, improved high toughness in combination with high hardness of steel can also be obtained through bainization or austempering. In this process the steel is also austenitized, but then instead of direct quenching to low temperatures, it is rapidly cooled to temperatures of around 300 °C to form bainite. This can be achieved by using a molten salt bath. However, the transformation requires longer times, so the isothermal holding times are typically around 2 h or more and depends on chosen composition, primary austenite grains size (PAGS), etc. The resulting bainite leads to the desired hardness, which is combined with particularly improved toughness and fatigue strength. After the austempering, the steel is cooled in air, during which some of the untransformed austenite can undergo the martensitic transformation.

Bainite forms through a military transformation [9], meaning there is practically no diffusion of iron and substitutional elements and only a limited diffusion of carbon. Bainite morphology can be roughly divided into lower and upper bainite. The lower bainite forms at lower temperatures and has carbides (orthorhombic Fe_3_C) within the bainitic ferrite needles, and is generally considered tough and hard. While the upper bainite is formed at higher temperatures, has carbides (hexagonal Fe_2.4_C) along the bainitic ferrite needles, and is considered hard and brittle [10]. With carefully chosen chemical composition the carbides can be suppressed to form carbide free ausferritic microstructure by austempering [11].

Therefore, the austempering temperature has a great influence on the bainitic transformation. The yield strength and toughness of the steel can be improved by increasing the bainitic ferrite volume fraction [12]. Although the morphology of the retained austenite can be coarser, the impact and fracture toughness may still be higher because of its higher bainite content. Compared to martensite, the main advantages of carbide-free bainite are the higher toughness and the high level of compressive stresses on the surface of the components. Additionally, the distortion values after austempering are lower in most cases, since smaller thermal and phase transformation stresses are generated. In was also shown by [13] that corrosion resistance of the high silicon austempered steel increased with increases in the volume fraction of retained austenite due to lower amounts of residual stresses.

The high silicon content in steels provides martensite stabilization and retards the formation of cementite and perlite during cooling, and therefore is also used in bainitic steels [14]. It is well known that silicon increases strength and hardness of martensite [15]. Use of silicon is also attractive due to the lower price.

The as-cast medium carbon high silicon steel described in this paper is used in rock breaking excavation application, so wear resistance is an important factor, along with hardness and impact toughness. In recent studies, researchers have focused more on the bainite transformations or mechanical properties and not so much on abrasion resistance of bainitic steels [16]. The tests performed on austempered high carbon, high silicon steel showed a drastic improvement in wear resistance [17], while medium carbon steels show mixed results, when studying abrasion wear resistance [18]. Therefore the aim of this study was to evaluate austempering instead of classic quenching heat treatment approach on wear resistance of as-cast medium-carbon high-silicon steel.

## 2. Materials and Methods

### 2.1. Material and Heat Treatment

As-cast medium-carbon low-alloyed steel with 0.32 wt.% C was used in this investigation. A low aluminum content was added to the steel to minimize the presence of harmful aluminate nonmetallic inclusions. The chemical composition is given in Table 1. The martensite start temperature for the steel composition was 295 °C. The chemical composition was determined using optical emission spectrometry (OES) and LECO for sulfur and carbon.

**Figure 1:**
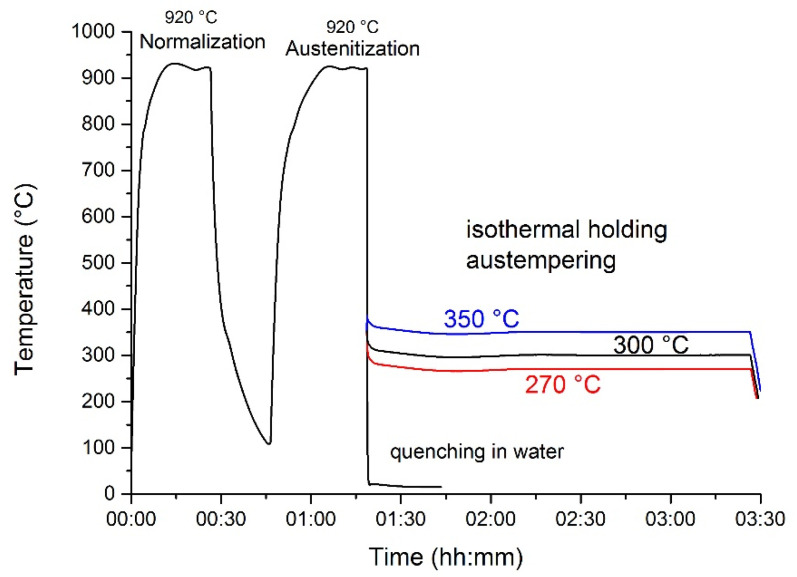
Graphical presentation of heat treatments.

The heat treatment conditions are summarized in Table 2. Graphical presentation of heat treatment can be seen in Figure 1. Three austempering heat treatments (samples B) and two conventional quenching treatments (samples Q) and were prepared. As it can be seen, all the investigated samples were firstly normalized at 920 °C and air cooled, then austenized at 900 °C and held for 15 min. The austempering samples were held in isothermal salt baths for 2 h at 270, 300 and 350 °C for sample B1, B2 and B3 respectively. The temperatures were chosen in order to attain lower and upper bainite. For the salt bath, a composition was chosen in respect to austenitization and isothermal holding temperatures, respectively.

Samples for conventional quenching treatments where quenched in water and then tempered at 180 °C for 2 h (sample Q1) or at 300 °C for 0.5 h (sample Q2).

### 2.2. Characterization

In order to investigate influence of heat treatment on microstructure, all samples were metallographically prepared by grinding with SiC papers, followed by polishing with 3 to 1 μm diamond suspension, and finally etching with 5 vol.% Nital for light optical microscopy (Olympus DP70 LOM). Microstructure and wear tracks were analyzed using scanning electron microscopy (ThermoFisher Scientific Quattro S field-emission SEM).

### 2.3. Hardness and Impact Toughness

In order to investigate the influence of different heat treatments on mechanical properties, Rockwell hardness and impact toughness were measure. Hardness on each type of sample was measured using Instron B2000 Rockwell test machine according to ISO 6508-1:2016 standard. Impact toughness was measured using the Charpy pendulum impact test performed according to SIST EN ISO 148-1:2016 standard, using standard Charpy V-notch test samples, and at −40 °C.

### 2.4. Tribological Testing

In order to evaluate influence of heat treatment on abrasive wear resistance reciprocating sliding using dry ball-on-flat contact configuration was used. Model wear tests were performed in order to eliminate other potential influences of environment on wear results. The test parameters and contact materials were selected so that the predominant wear mechanism was abrasion, as in real contact. Test parameters were also chosen to achieve measurable wear in a relatively short time.

All samples were polished (*S*a = 0.05 µm) before the test in order to minimize influence of roughness on tribological properties. To concentrate wear on the investigated steel samples, a much harder Al_2_O_3_ ball with diameter of 32 mm and hardness of 1250–1700 HV was used as a counterbody in sliding contact. Normal load of 61 N, corresponding to nominal Hertzian contact pressure of 1 GPa, was used. The frequency of reciprocating sliding was 1 Hz, corresponding to sliding speed of 0.008 m/s. Sliding time was limited to 1 h. At least three repetitions for each heat treatment type were performed in order to confirm repeatability. During testing, the coefficient of friction was recorded continuously. Coefficient of friction is defined by ratio of the frictional force resisting the motion of two surfaces in contact to the normal force pressing the two surfaces together. After the test wear volumes were measured using 3D profilometer Alicona Infinite Focus G4. Wear tracks were analyzed using optical and SEM microscope.

## 3. Results

### 3.1. Microstructure

**Figure 2:**
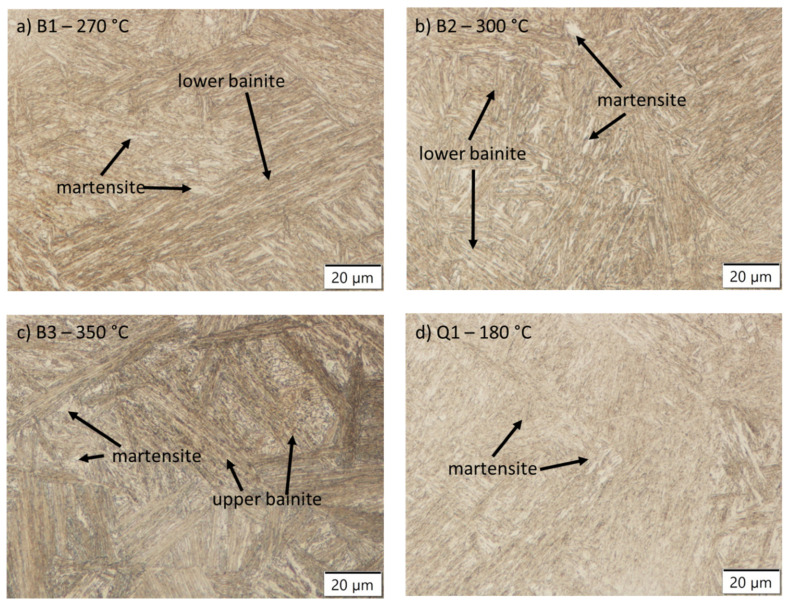

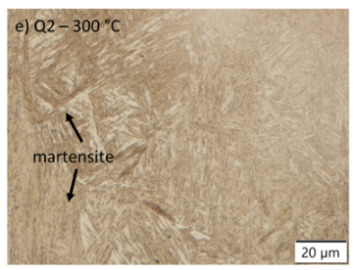
Optical microscopy of samples according to heat treatment cycle presented in Table 1: (**a**) sample B1, (**b**) sample B2, (**c**) sample B3, (**d**) sample Q1 and (**e**) sample Q2.

Light microscopy revealed that the different austempering temperatures resulted in three different microstructures. While the lowest austempering temperature of 270 °C (B1 sample) yielded a mixed microstructure of lower bainite and blocky martensite formed by cooling the sample under Ms (Figure 2a). The tempering effect of first nucleated martensite depended on the chosen temperatures. A microstructure of lower bainite and martensite was found also in sample B2 (Figure 2b), while the sample B3 has signs of upper bainite and martensite (Figure 2c). This is consistent with the findings of other researchers [10] that isothermal transformations under martensite start temperature (as in sample B1) results in a mixed type of bainitic-tempered martensitic structure. Both quenched samples Q1 and Q2 contained martensite; the difference was in the degree of tempering, sample Q1 was tempered at a low temperature (180 °C) for 2 h which mainly relieves stresses and modestly precipitates carbides. Sample Q2 had the typical structure of tempered martensite, which also more readily etches, with a more pronounced carbide precipitation.

**Figure 3:**
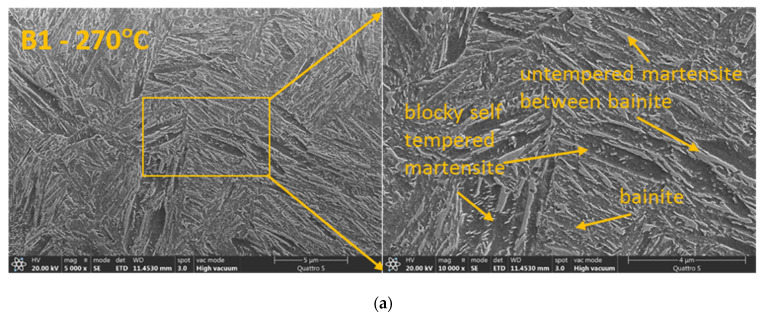

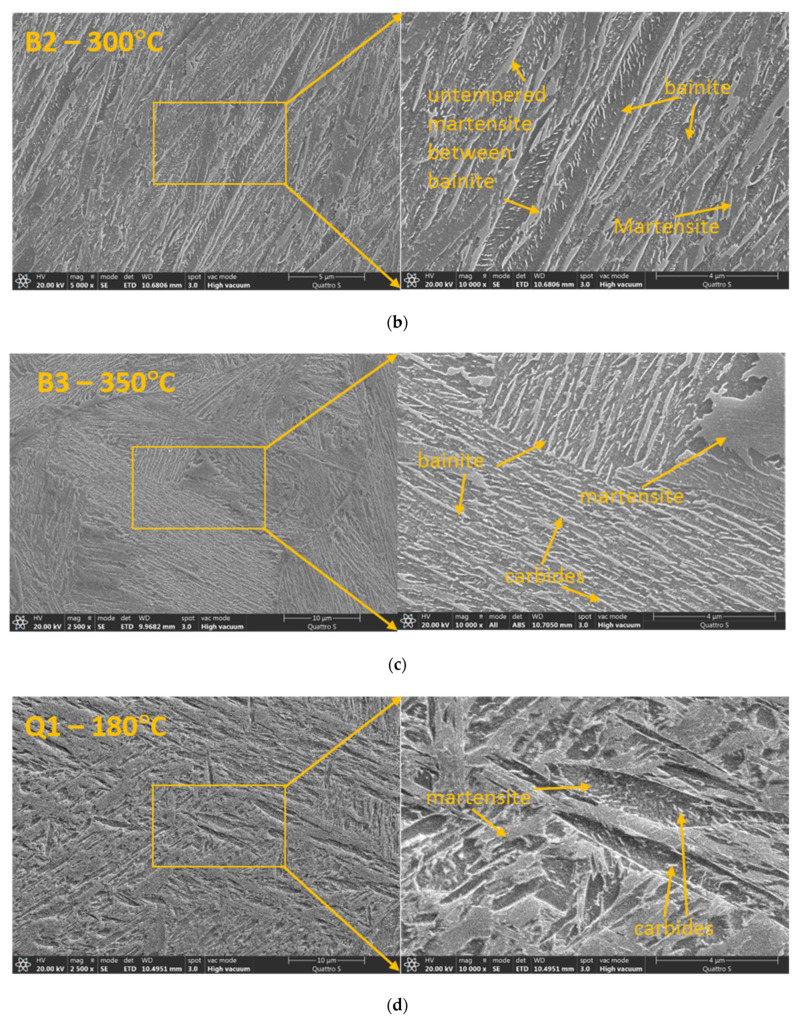

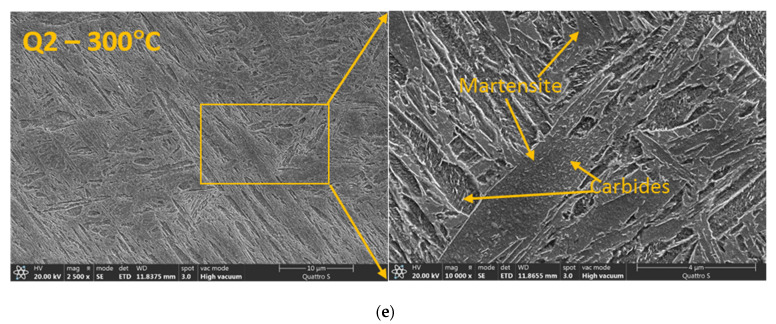
SEM of samples according to heat treatment cycle presented in Table 1: (**a**) sample B1, (**b**) sample B2, (**c**) sample B3, (**d**) sample Q1 and (**e**) sample Q2.

SEM investigation revealed more detailed insight into the microstructure. Bainite sheaves and untempered martensite formed between the sheaves along with coarse martensite are main characteristic observed in the austempered samples. As can be seen in Figure 3a the martensite obtained at 270 °C was different and closer to quenched and tempered state Q1 or Q2 (Figure 3d,e). The increase of the austempering temperature from 270 to 300 and 350 °C resulted in more heterogeneous mixed martensitic to bainitic microstructure. This is consistent with finding of Zhao et al. [19] where they pointed out the effect of carbon on the stability of retained austenite. The blocky untransformed austenite has an uneven distribution of carbon that results in martensite packets/laths of various sizes. The martensite blocks formed under M_s_ in sample B1 could be partially tempered (self-tempered) at the isothermal temperature (270 °C) due to high enough temperature to enable the precipitation of carbides and stress relieving. The austempering at 300 °C (B2) resulted in longer bainite needles than in sample B1. The presence of untempered martensite between bainite, formed during cooling after isothermal hold, was more evident (Figure 3b). The martensite between the bainitic was untempered, because it was the result of the martensitic transformation at a temperature that was lower that the isothermal holding temperature (300 °C) and rather intense cooling after isothermal holds. Similarly, sample B3 (350 °C) had even larger blocks of untempered martensite, but the bainite needles showed a clear pattern of carbide formation along the needles (Figure 3c). To sum up the results, the B1 sample was the only one that contained self-tempered martensite; all other austempered samples contained only untempered martensite among formed bainite.

Direct quenching resulted in fully formed lath martensite formation (Figure 2d). At lower holding temperatures (180 °C) carbides were longer and needle-like. With the increase of holding temperature carbides became less needle-shaped, but not yet spherical. At 350 °C (Q2), the martensitic showed large laths that formed first, with smaller laths that formed later at lower temperatures (Figure 3e).

### 3.2. Hardness and Impact Toughness

**Figure 4:**
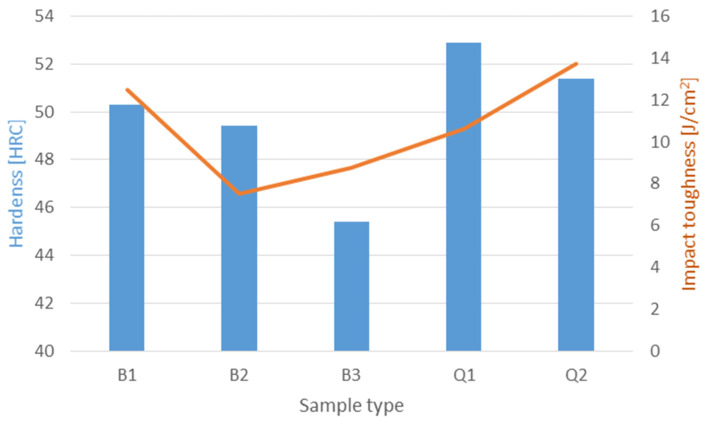
Hardness and impact toughness of investigated samples.

Figure 4 presents hardness and impact toughness values for the investigated samples. It can be seen that highest hardness values were obtained by quenched samples (Q1 = 52.9 HRC and Q2 = 51.4 HRC). On the other hand isothermal holding in a salt bath resulted in lower hardness values (From 50 to 45 HRC). Highest hardness in the austempered samples was at the holding temperature of 270 °C (50 HRC). The increase in the holding temperature to 350 °C lowered the hardness to 45 HRC. The decrease in hardness is related to the increase of blocky retained austenite remaining after partial martensite transformation and the coarsening of the microstructure [5].

The quenched and tempered sample Q1 had the highest hardness (53 HRC). This is associated with a low tempering temperature—180 °C. The higher tempering temperature—300 °C resulted in lower hardness (51 HRC). The hardness drop was limited due limited tempering time at 300 °C.

Different heat treatments also influenced the impact toughness. The highest value of impact toughness, 13.75 J/cm^2^, was obtained with quenched sample Q2. The combination of quenching and low temperature tempering in the Q1 sample resulted in a lower value of impact toughness—10.625 J/cm^2^. On the other hand austempering at the lowest temperature (B1) resulted in the second highest value of impact toughness—12.5 J/cm^2^. The increase of the holding temperature to 300 °C results in a 40% decrease in the impact toughness—7.5 J/cm^2^. On the other hand, further increase in the holding temperature to 350 °C resulted in a slight increase of the impact toughness—8.75 J/cm^2^.

Differences in mechanical properties can be attributed to the transformation of the unstable (low carbon), austenite to brittle martensite after continuous cooling to room temperature, or by mechanically (strain or stress) induced martensite transformation, the actual volume fraction of austenite and relative residual austenite stability. The so-called “blocky” austenite formed hard phases, not only untempered martensite, but also in some cases carbides. These can later create voids and lead to a decrease in toughness [20,21,22]. The overall hardness of a sample depends on the hardness and volume of all microstructural components, namely stable retained austenite, bainite (bainitic ferrite) and tempered or untempered martensite.

### 3.3. Tribological Testing

**Figure 5:**
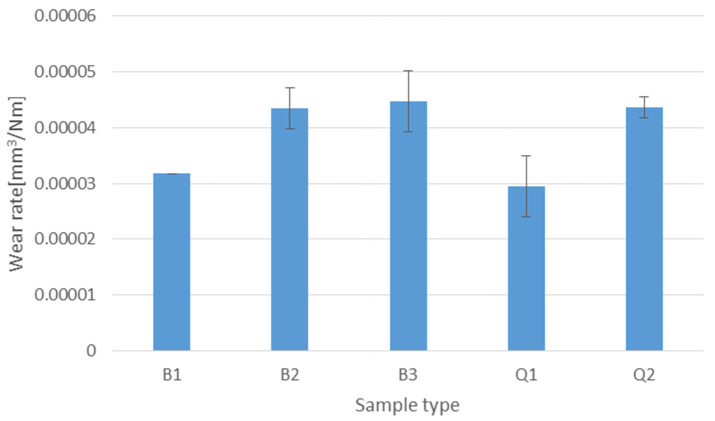
Wear rate of investigated samples.

**Figure 6:**
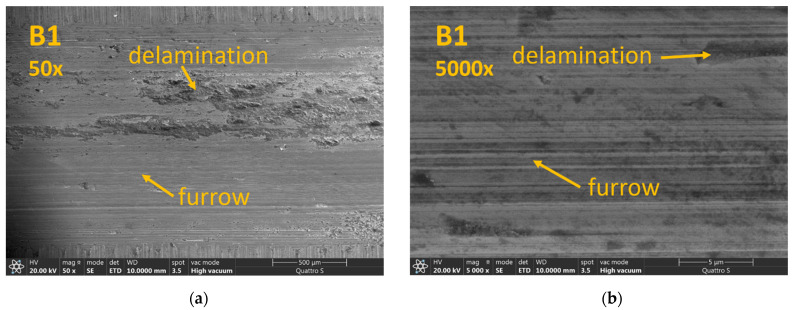

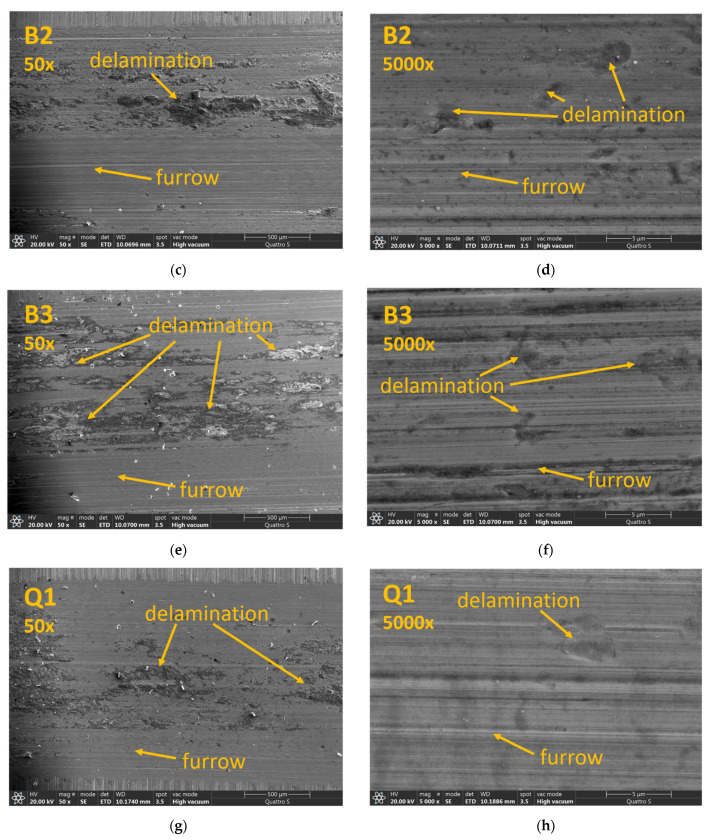

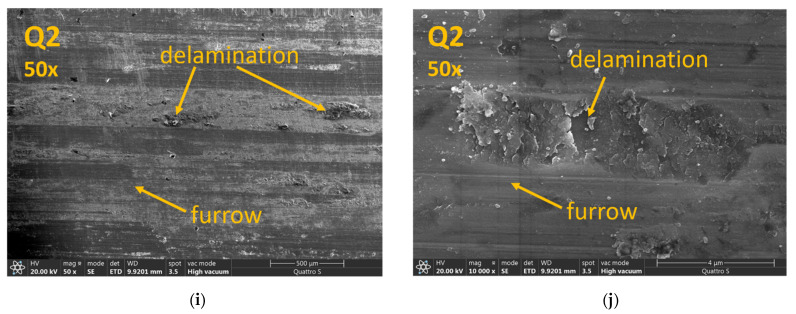
SEM of wear tracks at magnification 50× and 5000× for samples (**a**,**b**) B1, (**c**,**d**) B2, (**e**,**f**) B3, (**g**,**h**) Q1 and 50× and 10,000× for (**i**,**j**) Q2.

Results obtained from abrasive wear tests are shown in Figure 5. It can be seen that lowest wear rate was obtained for sample Q1 which also had the highest hardness. However, with sample B1 (lower bainite) which had lower hardness, comparable wear rate was obtained. Further, sample B1 also had 25% higher impact toughness in comparison to sample Q1. With already generally low impact toughness, such an increase can be crucial for practical applications. On the other hand, raising the austempering temperature to 300 or 350 °C had a negative effect on the wear rate. Although sample B3 had lower hardness than sample B2, their wear rates were comparable. Quenched sample Q2 had higher hardness than samples B2 and B3, but their wear rates were comparable. Results can also be correlated to the microstructures. As can be seen on Figure 2 sample B1 had relatively fine microstructure composed of lower bainite and martensite; on the other hand the microstructure of sample B3 showed the presence of coarse blocky martensitic islands and less fine bainite needles. Figure 6 shows SEM micrographs of worn surfaces. Since counter body in the wear test was much harder, it is obvious that the main wear mechanism was abrasion. For sample B1, a furrow morphology with areas of delamination can be seen (Figure 6a,b). Increase of temperature of isothermal holding in a salt bath from 270 to 350 °C resulted in increased of delamination areas in the wear track, with sample B3 resulting in a much higher number of delaminations and wear out particles when compared to B1 and B2 sample, respectively (Figure 6c–f). This can be contributed to the decrease in hardness (sample B3 had the lowest hardness among all investigated samples) and the different load-carrying capacity of bainite and martensitic blocks.

The main difference in microstructure between Q1 and Q2 was the presence degree of tempering. Figure 6i,j shows wear track of sample Q2 with detachment of larger martensitic blocks from the matrix. This can be also contributed to lower matrix hardness, because the more intensive tempering and hardness dropped. Although the overall hardness of quenched sample Q2 was higher than the austempered samples with bainitic microstructure, its wear rate was comparable to the B2 and B3 sample. This clearly indicates that hardness alone cannot be directly correlated with improved wear resistance and microstructure also plays an important part. Moreover, it shows the importance of properly chosen heat treatment parameters.

**Figure 7:**
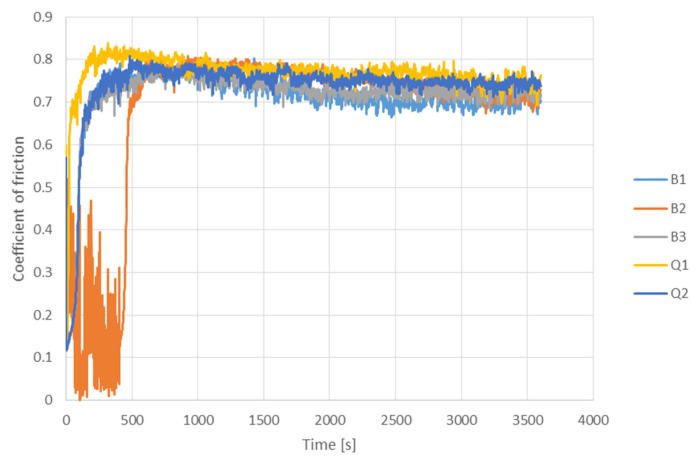
Coefficient of friction of investigated samples.

As can be seen in Figure 7, there was also a difference in values and distance to steady state of friction between samples. Lowest friction was obtained with sample B1 (0.71) and the highest with sample Q1 (0.77). Samples B2, B3 and Q2 on the other hand resulted in similar coefficient of friction values (0.73–0.74). The distance to steady state of friction was shortest for sample Q1 (250 s), when longest within sample B3 (480 s) which was also most restless at the beginning. Distance to steady state of friction for samples B1, B3 and Q2 were similar and in the range of 300 s to 400 s.

## 4. Conclusions

Based on the experimental work performed, the following conclusions can be drawn:

- In general, austempering resulted in lower hardness values when compared to classic quenching heat treatment (Q1 and Q2) with improved impact toughness when holding was performed under M_s_ for B1 taken as modified austempering step compared to other two austempering steps performed above M_s_ (B2 and B3).
- Modified austempering (B1) at 270 °C (below the martensite start temperature of 295 °C) resulted in the formation of mostly lower bainite and self-tempered martensite that gave good and comparable wear resistance to quenched and low temperature tempered (180 °C) sample Q1. The tempering of blocky martensite islands that form during austempering is crucial for higher wear resistance.
- Austempering where finer lower bainite was obtained resulted in higher hardness and impact toughness than where coarse upper bainite was obtained.
- Raising the austempering temperature to 300 or 350 °C had an obvious negative effect on the measured wear rate.
- Austempering the sample B1 at 270 °C achieved a comparable wear rate at lower hardness and improved toughness compared to quenched sample Q1 produced by classical water quench and tempering.
- Austempering the sample B3 at 350 °C resulted in overall decrease in wear and mechanical properties compared to lower temperature austemperings and quenching and low temperature tempered state.
- Comparable wear rate on samples produced by different thermal steps (B2, B3 and Q2) reveals that hardness is not a stable indicator of wear performance in as-cast wear resistant steels.

The presented paper shows that austempering has potential in practice on the given chemical composition inside a very narrow temperature region, indicating the possibility to use such treatments for highly complex as-cast parts where crack susceptibility is high due to nonuniform cross-section cooling.

## Figures and Tables

**Table 1:** Chemical composition of investigated as-cast steel grade (wt.%).

C	Si	P	S	Mn	Cr + Mo	Al	Ni	N	Fe
0.32	2.0	0.016	0.006	1.58	0.99	≤0.01	0.14	0.006	rest

**Table 2:** Heat treatment parameters for investigated samples.

	Normalization Temperature	Austenitization	Tempering	Isothermal Holding in the Salt Bath
B1	920 °C, 15 min, air	900 °C, 15 min	/	270 °C, 2 h
B2	920 °C, 15 min, air	900 °C, 15 min	/	300 °C, 2 h
B3	920 °C, 15 min, air	900 °C, 15 min	/	350 °C, 2 h
Q1	920 °C, 15 min, air	900 °C, 15 min, room temperature water	180 °C, 2 h	/
Q2	920 °C, 15 min, air	900 °C, 15 min, room temperature water	300 °C, 0.5 h	/

## Data Availability

Not applicable.
